# Integrating oral health into primary healthcare: lessons from project OHE-NCHeW (oral health education for nurses and community health workers) in Nigeria

**DOI:** 10.3389/froh.2025.1597243

**Published:** 2025-06-20

**Authors:** Abimbola M. Oladayo, Folake B. Lawal, Oyinkansola O. Sofola, Omolara G. Uti, Afolabi Oyapero, Adetayo Aborisade, Mojisola Olujitan, Omotayo F. Fagbule, Adeola T. Williams, Aderonke Dedeke, Ejiro Idiga, Yahya-Imam Abdul-Kabir Adegoke, Ilori Adeniji Oluwagbenga, Aishatu Baba Mele, Amina Sani Baffa, Ifeoluwa Adetula, Khadija Saad Musa, Bernal Stewart, Carlo Amorin Daep, Deon Hines, Jacinto Beard, Taiwo A. Lawal, Azeez Butali

**Affiliations:** ^1^ Missouri School of Dentistry and Oral Health, A.T. Still University, Kirksville, MO, United States; ^2^ Department of Periodontology and Community Dentistry, University of Ibadan and University College Hospital, Ibadan, Nigeria; ^3^ Fellow, Consortium for Advanced Research Training in Africa (CARTA), Nairobi, Kenya; ^4^ Department of Child Oral Health, University of Ibadan and University College Hospital, Ibadan, Nigeria; ^5^ Department of Preventive Dentistry, Faculty of Dentistry, Lagos State University College of Medicine, Lagos, Nigeria; ^6^ Department of Oral Diagnostic Sciences, Faculty of Dentistry, Bayero University Kano, Kano, Nigeria; ^7^ Department of Oral Diagnostic Sciences, Aminu Kano Teaching Hospital, Kano, Nigeria; ^8^ Department of Oral Pathology, Radiology and Medicine, College of Dentistry, University of Iowa, Iowa City, IA, United States; ^9^ Iowa Institute for Oral Health Research, University of Iowa, Iowa City, IA, United States; ^10^ School of Public Health, University of Nevada, Reno, NV, United States; ^11^ Department of Oral and Maxillofacial Surgery, Lagos University Teaching Hospital, Lagos, Nigeria; ^12^ Department of Restorative Dentistry, Lagos University Teaching Hospital, Lagos, Nigeria; ^13^ Department of Preventive Dentistry, Aminu Kano Teaching Hospital, Kano, Nigeria; ^14^ Faculty of Dentistry, Bayero University, Kano, Nigeria; ^15^ Colgate-Palmolive Company, Piscataway, NJ, United States; ^16^ National Dental Association Foundation, Washington, DC, United States; ^17^ Division of Pediatric Surgery, Department of Surgery, University of Ibadan and University College Hospital, Ibadan, Nigeria

**Keywords:** primary healthcare (PHC), oral health disparities, CREATIVE framework, nurses, community health worker (CHW), oral health education/promotion

## Abstract

**Introduction:**

Oral health disparities in Nigeria highlight the need to integrate basic oral health into Primary Health Care (PHC). Project OHE-NCHeW (Oral Health Education for Nurses and Community Health Workers) was developed to train PHC workers in providing basic oral health care, education, and referrals in underserved communities. This study explored the impact of the training program on participants' knowledge, referral practices, and patient outcomes, and gathered feedback to optimize the program for future implementation.

**Methods:**

Using a qualitative phenomenological approach, five focus group discussions (FGDs) were conducted with participants to gather insights on knowledge acquisition, changes in referral practices, and barriers or facilitators to implementation. Audio recordings were transcribed verbatim and analyzed thematically using the CREATIVE framework. Additional feedback from trainers and observed patient impact were also considered.

**Results:**

The trained PHC workers reported enhanced knowledge and confidence, resulting in improved patient education and referrals. Patients also noted better oral health literacy and increased trust in dental referrals. Facilitators viewed the program as valuable and feasible, emphasizing the importance of ongoing training, resource allocation, and policy integration to maintain the program's impact. Key barriers included insufficient resources, lack of supervision, and cultural beliefs.

**Discussion:**

The pilot training enhanced PHC worker capacity and was positively perceived by trainers. Patients reportedly responded favorably, indicating potential impact. However, addressing identified systemic and resource barriers is crucial for sustainable integration. This study offers multi-perspective insights for optimizing oral health integration within PHC in similar settings.

## Introduction

Oral diseases represent a significant global public health burden, impacting billions of individuals and contributing to substantial morbidity, pain, and economic costs (1). In Nigeria, Africa's most populous nation, oral health disparities are particularly acute, driven by factors including a severe shortage of dentists (estimated dentist-to-population ratio of 1:44,830 vs. WHO recommended 1:5,000) and their uneven distribution, with the majority concentrated in urban centers (2–4). This leaves vast populations, particularly in rural and underserved areas, with limited or no access to essential oral healthcare services (5).

Integrating oral health into Primary Health Care (PHC) is widely recognized as a cost-effective strategy to expand access to basic oral health services, promote prevention, and advance health equity, as advocated by the WHO (6–8). The existing PHC workforce in Nigeria, comprising nurses, community health workers (CHWs), and midwives, is well-positioned to deliver basic oral health interventions due to their reach within communities (9). However, previous studies in Nigeria and other settings have consistently reported inadequate oral health knowledge among these healthcare cadres (10–12) and have highlighted the need for structured training to equip them with the necessary skills and confidence (9, 13). Despite a willingness among some PHC workers to incorporate oral health into their duties, systemic barriers and a lack of adequate preparation remain challenges (11, 14, 15). To address this critical gap and strengthen the capacity of the Nigerian PHC workforce in oral health, Project OHE-NCHeW (Oral Health Education for Nurses and Community Health Workers) was developed (16). This comprehensive initiative designed a standardized, evidence-based curriculum and piloted its delivery among nurses and CHWs in three diverse states across two geopolitical zones in Nigeria. Unlike some previous, smaller-scale efforts, Project OHE-NCHeW aimed for a more standardized approach to facilitate broader implementation and evaluation (16).

To evaluate the sustainable potential of the Project OHE-NCHeW model for training PHC workers, it is vital to explore the views of major stakeholders about the training process. Moreover, stakeholders play critical roles in the success of educational programs (17, 18). Therefore, this specific manuscript presents a focused qualitative evaluation embedded within the Project OHE-NCHeW pilot phase. The primary objective of this study was to explore in-depth the experiences and perceptions of trained nurses and CHWs regarding their participation in the pilot training, their application of acquired knowledge and skills in practice, and the perceived barriers and facilitators encountered during the implementation of basic oral health interventions in their respective PHC settings. This qualitative exploration also incorporated feedback from trainers on the program's delivery and captured reported observations from patients regarding their encounter with trained PHC workers, providing a multi-stakeholder perspective on the feasibility and initial impact of integrating oral health through this training model. The findings from this study contribute crucial qualitative data to inform the refinement and future scaling of oral health training initiatives for PHC workers in Nigeria and similar low-resource contexts globally.

## Materials and methods

### Study design and setting

This study utilized a qualitative, phenomenological approach to evaluate the experiences and perceptions of the Nurses and CHWs who participated in the pilot training—Project OHE-NCHeW, in Lagos, Ibadan, and Kano, Nigeria, following project completion. We also reported on the experiences of the facilitators of the training and patients at the PHCs who benefited from the outcomes of the training. These locations were strategically selected due to their geographic, cultural, and socio-economic diversity, which provides valuable insights into the unique challenges faced by PHC workers across the country. Lagos, Nigeria's largest urban center, represents a metropolitan population with significant healthcare demand (19). Ibadan, the second-largest city in Nigeria, offers a mix of urban and peri-urban demographics, which provides a distinct perspective on regional healthcare access (20–22). Kano, in northern Nigeria, is characterized by its cultural richness and the need for tailored health interventions (23–25).

The pilot study employed a quasi-experimental design (pre- and post-with a non-equivalent control group) to assess the impact of the 5-day oral health training delivered on the knowledge level and referral practices of the participants (February to May 2024) (16). After completing the training, participants were asked to incorporate oral health education into their routine health promotion activities at their respective PHCs over 3 months. Full details of the training content and implementation process have been reported elsewhere (16). This study explores the perspectives of CHWs and nurses regarding the influence of the OHE-NCHeW training program on referral practices, knowledge dissemination, and clinical application using a qualitative approach. Additionally, insights were obtained from stakeholders and trainers to inform the refinement of training content and referral pathways. Patient interviews were also conducted across study sites to evaluate the perceived impact of the program from the service users' perspective, thereby contributing to a comprehensive assessment of program implementation and areas for optimization.

### Participants

The sample of trainees (nurses and CHWs) was drawn from a predefined “sampling frame” of all 60 participants (20 per study site) who participated in the 5-day pilot training (16). This virtual platform was created during the training to support and facilitate resource sharing among the trainers and research participants. From this group, 34 participants (about of the trained PHC workers) were randomly selected and took part in the FGDs. The eligibility criteria and complete description of the participant sampling have been described elsewhere (16).

Additionally, six members of the study team who were involved in implementing Project OHE-NCHEW were invited to participate in an unstructured interview. Thirty out of the 503 patients who were referred to the dental centers from the PHCs where the trainees worked and conducted oral health promotion activities were also engaged. The assessment of patients' perspectives aimed to understand how well the training was translated into practice and how it affected patient experiences and outcomes.

### Data collection

Data were collected using multiple qualitative methods to gain diverse perspectives on the pilot training. Course evaluation forms were administered immediately after the training to assess its impact and participant satisfaction (26). Completion of the evaluation form was voluntary and anonymous. For this manuscript, data derived from the open-ended questions on this form were utilized to provide qualitative insights into participants' experiences and suggestions (see Supplementary Material).

A total of five focus group discussions (FGDs) were conducted with the trainees at the host tertiary healthcare center, at the same site where the initial training occurred, to facilitate participant convenience and ensure robust participation (Table 1). These discussions included one in Lagos, two in Ibadan, and two in Kano, with 5–9 participants per group. Three licensed dentists facilitated the FGDs, consisting of two males and one female (AO, AO, and LFB), who had extensive research experience and were members of the study team. None of the facilitators had prior relationships with the participants or any conflicting interests or biases. The participants were assigned identification numbers to ensure anonymity and were compensated with a token equivalent to ∼$10 to cover transportation expenses to the interview site.

**Table 1:** Summary of focus group discussions (FGDs).

FGD No.	Participant type	No. of participants	Mode
FGD 1 (Lagos)	Nurses and CHWs	7	Physical
FGD 2 (Ibadan)	Nurses and CHWs	9	Physical
FGD 3 (Ibadan)	Nurses and CHWs	8	Physical
FGD 4 (Kano)	Nurses and CHWs	5	Physical
FGD 5 (Kano)	Nurses and CHWs	5	Physical

Each FGD lasted approximately 2 h and was audio-recorded with participants' consent. The discussions were guided by semi-structured interview guides, which were developed collaboratively by the research team based on the training objectives and a review of relevant literature (see Supplementary Material). The guides were tailored to assess the perceived impact of the training, identify areas for improvement, and explore the feasibility and sustainability of integrating oral health promotion into routine maternal and child health (MCH) services. Versions of the guides were adapted for linguistic and cultural relevance and translated into local languages (Hausa, Yoruba, and Fulfulde) to ensure participant comprehension and engagement. Complex terms were summarized and interpreted in local languages during the sessions. Although audio recordings were made for transcription and analysis, no field notes were taken during the discussions.

Additionally, an unstructured interview was conducted with six facilitators who delivered the pilot training immediately after the training sessions and was audio-recorded. The face-to-face interview, guided by a set of open-ended questions (see Supplementary Material), was largely conversational and aimed to capture the facilitators' experiences, challenges, and perspectives on the program's delivery and perceived impact.

Lastly, initial feedback was sought from a sample of patients who benefited from oral health promotion activities and required referrals following their interaction with trained PHC workers. This feedback was collected through phone calls made by trained project staff using a standardized phone script (see Supplementary Material). These calls aimed to capture patients' immediate perceptions of the oral health information or referrals received. While the script included some closed-ended questions, the primary goal of this evaluation was to gather qualitative insights, and the data collection method was not designed for rigorous quantitative analysis of patient outcomes.

### Data analysis

Qualitative data analysis was conducted separately for each distinct data source. The qualitative data collected from the open-ended questions on the course evaluation forms were analyzed using a descriptive approach to summarize frequently mentioned comments and suggestions.

The transcripts obtained from the FGD audio recordings were transcribed verbatim and checked by two members of the research team. Analysis was done using the CREATIVE approach, adapted from Pitney and Parker (27). This framework involves the following steps: Considering the research questions and study purpose; Reading through transcripts to develop a comprehensive understanding of the data; Examining the data for significant insights related to the research questions; Assigning descriptive labels to data units to capture their essence; Thematizing the data to organize recurring patterns; Interpreting the themes in relation to the study's objectives; Verifying the findings to ensure accuracy and credibility; and Engaging in the writing process to present the analyzed findings systematically (27). Discrepancies in coding were resolved through team discussions (28). Trustworthiness was enhanced through prolonged engagement in the study settings to understand the context and participants, and comprehensive descriptions to provide detailed accounts that support the transferability of findings (29).

The audio recording of the unstructured interview with the trainers was transcribed verbatim. The transcripts were then reviewed to identify recurring perspectives and significant insights shared by the facilitators regarding their training delivery experience. Responses collected via the patient feedback phone script were reviewed to identify recurring perceptions and notable points from patients. Findings derived from the analysis of each of these data sources (PHC worker FGDs, trainer interviews, and patient feedback) were analyzed and are reported separately in the Results section to present the distinct perspectives of each stakeholder group on the Project OHE-NCHeW pilot training and its initial impact.

### Ethical considerations

Prior to their involvement in the qualitative study, informed consent was obtained from all participants, including trainers, trainees (nurses and CHWs), and patients. Ethical approval for the study was obtained from the Institutional Review Boards at the University of Iowa (IRB-01/202312517); the University of Ibadan/University College Hospital, Ibadan (UI/EC/23/0724); the University of Lagos/Lagos University Teaching Hospital, Lagos (ADM/DSCST/HREC/APP/6454) and Bayero University Kano/Aminu Kano Teaching Hospital, Kano (NHREC/28/01/2020/AKTH/EC/3739), Nigeria.

## Results

### Summary of the course evaluation responses

Post-course evaluation feedback from trainees highlighted the training's strengths and areas for improvement. Participants appreciated the training's detailed, practical content, the trainers' expertise, and the interactive format featuring Q&A sessions and outcome-focused quizzes. They praised the simplicity and clarity of the materials, the availability of reference modules, and the effective communication methods.

To enhance future sessions, participants suggested more frequent training (quarterly or biannually), expanding the program to include traditional birth attendants (TBAs), voluntary health workers, nursing administrators, and community health officers. They recommended incorporating practical demonstrations at dental clinics, offering residential training formats, increasing the use of teaching aids, and extending the curriculum to cover broader aspects of dental care. Participants also emphasized raising program awareness via social media and expanding the program's reach into communities. This feedback affirms the program's impact while identifying opportunities for refinement to maximize its reach and effectiveness.

### Focus group discussion with trainees

We identified five themes that summarized the participants' perspectives regarding the recently completed training (Table 2); (1) Knowledge and Skill Acquisition, demonstrating improved personal and professional understanding of oral health; (2) Practical Application, showcasing real-world implementation in clinical and community settings; (3) Challenges in Implementation, identifying barriers such as patient misconceptions and resource limitations; (4) Sustaining Oral Health Initiatives, offering recommendations for program sustainability; and (5) Monitoring and Future Directions, emphasizing the need for continued evaluation and community engagement. The table below presents these themes alongside derived subthemes and illustrative quotes, providing a structured overview of participants' experiences and insights.

**Table 2:** Themes and subthemes from FGD with trained PHC workers.

Theme	Subtheme	Illustrative quote
Knowledge and skill acquisition	Personal knowledge improvement	“*So, concerning the training we had three months ago, it was very effective and it is a life-changing training because there are some things even I did not know about oral health*.” – P3 Lagos 1. “*I learned how to use normal techniques of brushing and the size of toothpaste that we use*.” – P4 Lagos 1.
	Professional skill enhancement	“*…you know this training has helped a lot because it has changed our orientation about oral health, and it is now part of our daily health education*.”– P4 Active IB2.
	Behavioral change	“*This training you gave us made us all proactive in taking care of our mouths; it is an eye-opener for everybody*.” – P5 Active IB1.
	Knowledge dissemination	“*We have been successful in imparting new knowledge to our students, pregnant women, and many other people in the community, including our own families*.” – P1 Kano 2.
Practical application	Clinical skills (examination & referral)	“*We examine for tongue tie, we examine for oral health problems, and every day we examine their mouth, we direct them, tell them what to do*…” – P4 Lagos 1. “*Before the training, I was unable to identify some of the oral medical conditions. But during the training, I was able to identify this as oral hygiene, this is cleft lip, this is cleft palate*.” – P5 Lagos 1.
	Patient education & counseling	“*I just talked to women about morning sickness, if you are experiencing it, you do not brush immediately, you use sodium bicarbonate and water to rinse your mouth…*” – P4 Lagos 2.
	Community outreach	“*I have been passing the information to all my people, including my church members. Also, in my center during my ANC clinic, I tell all the pregnant women*…” – P3 Active Ibadan 1. “*I taught my wife how to take care of her oral cavity. We collectively taught our children*.” – P1 Kano 2. “*When I go to the class to educate the pupils, I go with the flyers, the model that you gave us, and the sachet toothpaste that you gave us, and all that, and they are taking it home. They take it home to their family and talk to them about their oral health*” – P2 Lagos 2.
Challenges in implementation	Patient reluctance & misconceptions	“*A patient told me it is better to clean her mouth with her fingers than use a toothbrush*.” – P6 Kano 1. “*During immunization, most of the mothers used to give the babies sweets and sugary things, and I tried to convince them to stop it in order not to let them have caries*.” – P1 Active IB1.
	Resource & logistical constraints	“*Most people that come to health centers have no money, so immediately when we mention referral to them, they will reject it*.” – P2 Active Ibadan 1. “*You have two people on duty; on antenatal day, we are the ones doing the examination, the test, and the lab work; we are the ones doing it out-patient. The workload at the PHC is too much*” – P1 Ibadan 2.
	Need for continuous training	“*Consistent training, like now, over three months that we have been training, even if it is six months because I feel like I am not fully trained…*” – P4 Ibadan 1B Active.
Sustaining oral health initiatives	Resource allocation & infrastructure	“*Another way to sustain this initiative is by providing materials for oral examinations as they are not available in PHCs*.” – P2 Kano 2. “*The best thing is for us to have an oral clinic in our health centers. We have a lot of space in our center. We can create space for you. We will need you 100%; we have a lot of patients there*.” – P2 Ibadan Active 1A.
	Policy & institutional support	“*Involving the leaders of the local government, that is, the medical officer of health in charge of the local government*…” – P1 Lagos 1.
	Training expansion	“*The training needs to be incorporated into the curriculum of the community health workers and the community nursing*.” – P1 Kano 2. “*It is sustainable if they can continue training and follow up with patients they report, as well as train more health workers because just a few people cannot do it*.” – P5 Lagos 1.
Monitoring & future directions	Regular supervision & follow-up	“*The monitoring and evaluation should be carried out by the trainers regularly, maybe at a three-month interval*…” – P1 Kano 2.
	Community engagement & awareness	“*By continuing the oral health education at the PHCs, conducting oral examinations, and educating clients on common oral dental diseases*.” – P3 Kano 2.
	Media & public campaigns	“*Another way of sustaining this initiative is using anchored radio and television programs to educate people with practical demonstrations constantly*.” – P1 Kano 2.

#### Feedback from trainers

Overall, trainers described their experiences as positive, highlighting high trainee responsiveness and eagerness to apply their knowledge in practice. Speaker P2 noted, “*The interactiveness of the students; the whole point of these lectures is to see that not only were they interested, but they were also interested in coming and using it in interactions with their clients*.” Trainers emphasized the value of pre-implementation activities, such as tailoring content to the local context through culturally relevant examples and adapting materials from the WHO manual. Speaker P3 explained, “*The first thing was to relate the topic to what they have done initially and what they have on the ground in their centers. So, based on that, we are able to tailor the lecture to suit what they are already doing*.”

Effective elements of the program included interactive sessions, local songs, familiar scenarios, and visual teaching aids. Speaker P2 shared, “*The activities, I think the activities…and even the pictures. A lot of the modules had pictures so that they could see*.” Speaker P4 added, “*I think one of the improvements I noticed was relating those pictures to cases that they had seen before, and they had most likely mismanaged*.” Challenges included the need for more visual aids and practical tools, as mentioned by Speaker P1: “*I felt I needed more pictures so that they could see the cases more clearly. To overcome this, we downloaded pictures and shared them with the participants using mobile tablets*.” Adapting to local challenges, such as finding alternatives for infant oral hygiene, demonstrated the importance of context-specific solutions.

Trainers observed significant impacts of the program, including improvements in trainees' oral health practices and their ability to link lessons across modules. Speaker P3 highlighted the importance of follow-up support, suggesting, “*If they do not have the support of their direct superiors, there is little they cannot actually do on their own*.” Proposed enhancements included creating a WhatsApp group for reminders and incorporating oral health into the broader healthcare system to address staffing shortages.

### Assessment of oral health education impact on patients attending PHCs

Patient perspectives on the oral health education and referrals provided by the trained PHC workers were gathered through follow-up phone calls. Most patients expressed satisfaction with PHC workers, reporting they received clear and comprehensive information about visiting dental centers. This contributed to high referral follow-through, reflecting the training's effectiveness. Patients overwhelmingly described the information provided by PHC workers as clear or very clear, which enhanced their understanding of what to expect at dental centers and increased trust. The principal themes and illustrative points derived from this patient feedback are presented in Table 3.

**Table 3:** Themes and subthemes from patient feedback.

Theme	Subthemes	Illustrative quotes
Communication and understanding	Clear and comprehensive education from PHC workers	“*She explained very well, I understood the basics of oral hygiene*” – P4 Kano. “*They are good with their work, and they made us understand the role of oral health to overall health*” – P12 Kano.
Barriers to follow-up	Perceived lack of immediate need (asymptomatic)	“*Because I was not in pain*” – P2 Kano.
	Financial constraints and clinic fees	“*No, I do not have money*” – P4 Kano, P8 Kano, P2 Lagos. “*I was told to pay before obtaining a card, and I do not have money. We get free services at the PHC*” – P4 Lagos.
	Traumatic past experiences and cultural discouragement	“*Even though my in-laws stopped me because they had an awful experience with tooth extraction*” – P6 Kano.
	Inaccessibility of referral centers, especially for rural patients	“*People in rural areas cannot afford to visit referral centers that are far from their area of residence. I suggest the provision of dental clinics in accessible areas*” – P9 Kano.
Suggestions for improvement	Establish accessible and well-equipped dental centers	"*Bring in more equipment to meet the needs of the ever-increasing number of patients*.” – P1 Kano. “*Provision of well-equipped dental clinics in other rural areas instead of referral to centers that are very far from one's residence*” –P7 Kano.
	Integrate dental services into PHCs	“*I suggest they should provide dental service at the PHC level so that people do not have to go far for treatment*.” – P3 Lagos. “*Provision of dental units in PHCs since they are closer to people*,” – P1 Kano.
	Improve service delivery at referral centers	“*After being referred from the center, the patient should be seen directly*”– P6 Kano. “*We were promptly attended to at the PHC, but there was a delay at the dental center*” – P8 Lagos.
Program impact	Improved patient awareness and education	“*I suggest this kind of health talk should be done more frequently, especially to rural dwellers, because the oral health talk I listened to was the first of its kind I have ever received. I believe if extended beyond the walls of the hospital, many people will benefit, and dental diseases will drastically reduce*”. – P15 Kano.

## Discussion

This qualitative study provides valuable insights into the feasibility and challenges of integrating basic oral health services into Primary Health Care (PHC) in Nigeria through the pilot implementation of the Project OHE-NCHeW training program. The study focused on task shifting, a leading strategy where tasks are transferred from highly qualified healthcare professionals to CHWs and nurses to address healthcare worker shortages and improve access to services (30). By capturing multi-stakeholder perspectives from trained PHC workers, trainers, and initial patient feedback, our findings highlight the program's immediate successes, the persistent systemic barriers, and critical considerations for future scale-up and sustainability.

### Enhanced knowledge acquisition and practical application at the PHC level

Study findings indicate that the Project OHE-NCHeW training effectively enhanced the oral health knowledge and skills of participating nurses and CHWs. PHC workers reported a significant shift in their orientation towards oral health, describing the training as an “eye-opener” and leading to behavioral changes in their practices. While the training was for all participating PHC workers, some of the most prominent examples of practical application emerged from their work with expectant mothers and young children. This aligns with previous studies demonstrating that targeted training can significantly improve the oral health knowledge of non-dental healthcare professionals, especially in low-resource settings where dental professionals are scarce (15, 31–36). The acquisition of skills, such as identifying oral conditions and providing basic oral hygiene education, empowered these workers to actively integrate oral health into their routine activities. This newfound awareness was also extended to community outreach, with participants actively disseminating oral health information in markets, churches, and schools.

In some cases, trainees educated family members and church congregations, thereby amplifying the reach of the training beyond their immediate work environment. This direct application of acquired knowledge, evidenced by workers actively using flyers, models, and even personal examples in their education efforts, highlights the immense potential of a well-trained PHC workforce to bridge critical gaps in oral healthcare access, consistent with the World Health Organization's advocacy for oral health integration into PHC (7, 37). Oral health education programs for non-dental practitioners have been successful in many settings, improving confidence in examining the mouth (35, 38) and referring to oral health providers (36). The universally acknowledged contribution of PHC workers to healthcare is entrenched in policy statements and related research that recognize them as a key link between professional care providers, such as physicians and patients (36, 39, 40). The USAID Health Care Improvement Project suggests that CHWs should receive ongoing training every 6 months to develop new skills, reinforce pre-service training, and ensure prior skills are being properly practiced (41). Informing non-dental healthcare workers about the intersection of oral care with medicine and providing them with clear guidance on how they can protect and promote patients' oral health may facilitate consideration of oral health in their practice (35, 36).

### Insights from trainers and patient experiences

The perspectives of the trainers further validated the program's design and delivery, with trainers perceiving the modules as valuable and feasible for PHC workers, which is crucial for successful program implementation (42, 43). Patient feedback, collected through phone calls, corroborated the effectiveness of the PHC workers' enhanced communication skills. Most patients reported receiving clear and comprehensive information, which enhanced their understanding and contributed to reported referral follow-through. This positive patient reception is critical, as patient understanding and acceptance are key determinants of health intervention success (44).

### Persistent barriers to sustainable integration

Despite these successes, our study identified significant barriers to the sustained implementation of oral health services, mirroring challenges reported in similar initiatives in developing countries (45, 46). Financial constraints emerged as a significant deterrent for patients, who often could not afford transportation to referral centers or the fees associated with specialist dental care, echoing findings across the globe (47–49). Thus, integrating more affordable or subsidized dental services within PHCs could improve affordability and care access. Patient misconceptions and reluctance to adopt new practices also presented challenges (50–54). This highlights the broader challenges in altering patient attitudes toward preventive care, which should be a central focus for future health promotion interventions. Logistical challenges within the PHC settings, including staff shortages, heavy workloads, and insufficient basic resources, were frequently cited by PHC workers. These resource limitations are commonly reported obstacles to PHC strengthening in Nigeria and other low-income countries, impeding the effective integration of new services (55–58). Furthermore, negative past experiences (59, 60) and rural accessibility issues further hindered follow-up care. Thus, while the training program successfully improved patient education and referrals at PHCs, addressing structural barriers such as cost, accessibility, and patient perceptions will be essential for sustaining the program's impact.

### Recommendations for sustaining and scaling oral health initiatives

The suggestions from PHC workers and patients offer a roadmap for optimizing the Project OHE-NCHeW model and similar future initiatives. Participants' recommendations for allocating dedicated resources for oral examinations at PHCs, establishing specialized oral clinics within existing PHC facilities, and simplifying referral processes are aligned with calls for strengthening PHC infrastructure and capacity (61–63) The emphasis on continuous training, workforce expansion to include other cadres like Traditional Birth Attendants (TBAs), and the integration of oral health into existing curricula are crucial for building a sustainable oral health workforce, a strategy supported by studies on task-shifting and health worker capacity building (4, 64, 65). Furthermore, suggestions for institutional support, policy integration, and robust monitoring and evaluation mechanisms highlight the critical role of health system governance and multi-sectoral collaboration for successful program sustainability (66, 67). The proposal for public awareness campaigns through various media and the provision of incentives for patients reflects strategies known to enhance patient engagement and uptake of health services in low-resource contexts (68, 69). Furthermore, a problematic referral system, characterized by feedback gaps and cumbersome processes, undermined the continuum of care, a barrier also documented in broader health system strengthening efforts (70, 71). This highlights the need for improved coordination between PHC and secondary care levels to ensure seamless patient journeys and maximize the impact of referrals. The need for continuous training and re-training, voiced by PHC workers who felt the initial training was insufficient to achieve complete competence, underscores the importance of ongoing professional development and supportive supervision for new skill adoption (72).

### Strengths and limitations

A key strength of the training is its comprehensive and interactive approach. Participants consistently noted that the use of case studies, images, and quizzes helped reinforce their learning. Trainers effectively adapted the content to the local context, making the material more relatable and actionable. Additionally, the program's structure, which combines theoretical lessons with practical applications, enables trainees to develop a solid understanding of key oral health concepts. These strengths were further reflected in the participants' ability to apply their new knowledge to patient interactions and community education. Furthermore, unlike other studies, which focused predominantly on urban populations or PHC workers from only one state or subregion (15, 33), our sampling strategy enabled the recruitment of a more diverse geographic distribution of PHC workers from three states in Nigeria from both the northern and southwestern regions of the country. The inclusion of expanded modules, culturally relevant materials, and case scenarios also distinguishes this program from previous ones.

Our study's qualitative design, while providing rich, in-depth perspectives from multiple stakeholders, inherently limits the direct generalizability of findings beyond the specific pilot sites in Nigeria. The findings represent the perceptions of participants at a specific point in time, and long-term outcomes regarding sustained practice changes among PHC workers and direct patient health improvements were not within the scope of this qualitative evaluation. Furthermore, the patient feedback was collected via a convenience sample of phone calls, which might introduce selection bias. Future research should include quantitative measures of impact (e.g., changes in oral health status, referral completion rates), long-term follow-up, and explore the generalizability of these findings across diverse geographical and socio-economic contexts within Nigeria.

### Implications for practice and policy

Health ministries and policymakers should consider allocating more resources for oral health training programs, especially in underserved areas. The program's success could be scaled up by leveraging trained PHC workers to train peers. Moreover, in light of the anticipated increase in awareness and oral health-seeking behavior from patients, resulting from the program, the current dental care financing mechanisms in low- and middle-income countries that inadequately shield the public from the economic burden of dental treatment should transition more, from out-of-pocket payments to prepayment and risk-pooling systems to better protect households from significant health expenses and reduce the financial strain of healthcare costs.

## Conclusion

The Project OHE-NCHeW pilot training improved PHC workers' oral health knowledge and patient referrals. Despite positive feedback, systemic challenges—costs, resources, and referral gaps—must be addressed for long-term success. The multi-stakeholder insights gained from this qualitative evaluation provide actionable recommendations essential for optimizing the impact and scalability of oral health integration initiatives within Nigeria's broader public health framework, ultimately contributing to better oral health outcomes and overall well-being in underserved communities.

## Data Availability

The raw data supporting the conclusions of this article will be made available by the authors, without undue reservation.
