# Supplementary material for: Integrating oral health into primary healthcare: lessons from project OHE-NCHeW (oral health education for nurses and community health workers) in Nigeria
Source: Front Oral Health. 2025 Jun 20;6:1597243. doi: 10.3389/froh.2025.1597243 (PMC12226467; doi:10.3389/froh.2025.1597243)
Supplement: Supplementary file 1 [file Table1.docx]

**COURSE EVALUATION**

Thank you for your participation in the Oral Health Education for Nurses and Community Health Workers training. To assess the impact of the training program and satisfaction of trainees, please complete this form in its entirety.

Please be honest in your assessments and answer the questions as fully as possible. Your feedback will be carefully reviewed and used by the course organizers to inform future training and curriculum adjustments, as needed.

|  | Yes | Somewhat | No |
| --- | --- | --- | --- |
| Were the training objectives clearly defined? |  |  |  |
| Were the topics covered relevant to you? |  |  |  |
| Did the training meet your expectations? |  |  |  |
| Was the content well organized & easy to follow? |  |  |  |
| Was the content structure clear and logical? |  |  |  |
| Did the trainer provide relevant examples during the training? |  |  |  |
| Was the trainer able to answer all your questions and concerns? |  |  |  |
| Did you have enough time allocated to complete the training? |  |  |  |
| Was the training interactive enough? |  |  |  |
| Were participation and interaction encouraged? |  |  |  |
| Were the materials distributed helpful? |  |  |  |
| Did the multimedia used within the training make it  easier to comprehend the topic? |  |  |  |
|  |  |  |  |
|  | **Good** | **Neutral** | **Needs Improvement** |
| What do you think of the trainer’s proficiency? |  |  |  |
| How would you describe the training pace? |  |  |  |
| How would you rate the training overall? |  |  |  |
| How well was the training structured? |  |  |  |
|  |  |  |  |
|  |  |  |  |
|  | **Yes** | **Maybe** | **No** |
| Would you recommend this training to a friend or colleague? |  |  |  |

What did you like the most about the training?

What would you change about the training?

How could this training be improved?

| **Likes** | **Proposed Changes** | **Proposed Areas of Improvement** |
| --- | --- | --- |
| Detailed, practical and educative | Increased training frequency | Residential vs Non-Residential |
| Trainer experience and expertise | Widen the reach in the community - TBA & Voluntary Health Workers | More teaching aids |
| Participatory aspect-Q&A sessions, outcome-oriented quizzes and practical examples | Integration of nursing admin and CHOs who can provide more guidance and supervision post completion | Extend training reach into the communities |
| Simplicity of training content | Visit to dental clinics for real life demonstration | More time for training |
| Modules for reference purposes |  | Include a broader aspect of dental care |
| Mode and channel of communication |  | Increase the awareness about the program and its outcomes on social media. |
