# Supplementary material for: Integrating oral health into primary healthcare: lessons from project OHE-NCHeW (oral health education for nurses and community health workers) in Nigeria
Source: Front Oral Health. 2025 Jun 20;6:1597243. doi: 10.3389/froh.2025.1597243 (PMC12226467; doi:10.3389/froh.2025.1597243)
Supplement: Supplementary file 2 [file Table3.docx]

INTERACTION GUIDE WITH SELECT PATIENTS

1. General Experience and Satisfaction

- Did you visit the dental center after being referred by the PHC worker?
- If no, why not?

1. Quality of Information Provided

- How clearly did the PHC worker explain why you needed to visit the dental center?
  - Very clear, Clear, Somewhat clear, Not clear at all
- What information did the PHC worker provide about the dental center and the services available?
- Did you feel well-informed about what to expect at the dental center before your visit?
- If no, what information was missing?

1. Feedback on PHC workers

- How would you rate the PHC worker's knowledge about dental health issues?
  - Very knowledgeable, Knowledgeable, Somewhat knowledgeable, Not knowledgeable
- Did the PHC worker provide you with helpful tips or advice on maintaining oral health?
- If yes, what tips or advice were provided?

1. Additional Comments

- Do you have any suggestions for improving the referral process or the services provided at the dental center?
- Is there anything else you would like to share about your experience with the PHC worker or the dental center?
