# Supplementary material for: Integrating oral health into primary healthcare: lessons from project OHE-NCHeW (oral health education for nurses and community health workers) in Nigeria
Source: Front Oral Health. 2025 Jun 20;6:1597243. doi: 10.3389/froh.2025.1597243 (PMC12226467; doi:10.3389/froh.2025.1597243)
Supplement: Supplementary file 3 [file Table4.docx]

**Unstructured Interview Guide**

1. What were the highlights for you in delivering this training? What stood out for you?
2. How did you tailor the training to meet the specific needs of your audience, considering the diverse backgrounds and experiences of the trainees?

1. In your opinion, what were the most effective elements of the training modules in terms of knowledge retention and application in real-world settings?
2. Can you share any challenges you encountered during the training sessions? How did you address or overcome them?
3. How did you foster engagement and participation among the trainees? Were there any particular strategies or activities that proved especially successful?
4. What feedback did you receive from trainees regarding the relevance and applicability of the oral health training modules?
5. What additional support or resources would enhance the effectiveness of future oral health training initiatives for healthcare professionals, nurses, and community health workers?
6. In the long term, do you envision a community health worker training program (e.g., year one or two) incorporating oral health training as a standard component?
