# Supplementary material for: Integrating oral health into primary healthcare: lessons from project OHE-NCHeW (oral health education for nurses and community health workers) in Nigeria
Source: Front Oral Health. 2025 Jun 20;6:1597243. doi: 10.3389/froh.2025.1597243 (PMC12226467; doi:10.3389/froh.2025.1597243)
Supplement: Supplementary file 4 [file Table5.docx]

**FOCUS GROUP DISCUSSION**

**Aim of the Focus Group**: The qualitative assessment to assess the **impact of the training,** **points of improvement** and the **practicability of incorporation** of oral health promotion activities into maternal and child health visits will be conducted with select participants in the intervention group at the 3-month evaluation using Deliberative Focus group discussions; this approach to evaluation serves to enhance program feedback through participants' experiences and opinions.

**Training Impact**

1. Can you share your thoughts on the oral health training you received?
2. What new knowledge or skills have you gained from the training ?
3. Since you completed the OHE-NCHEW training, have you observed any changes in your approach mother and child health?

- If yes, can you provide specific examples of how the training has influenced your practice?
- If no,why?

**Points of Improvement for project OHE-NCHEW**

1. Were there any aspects of the training module that you found enjoyable and suited your educational needs?

Prompt participants to provide more information.

- Which ones
- In what ways or how?

1. Did you find any aspects of the training module particularly challenging or unclear?

If yes, which aspect.

- How were they/ is it challenging?
- Were you able to overcome the challenge/ challenges?
- If No, how could we have trained you better and what could we have done to address the problem?
- If yes, how were you able to overcome the challenges?

1. In your opinion, would you say that exposure to the training has adequately prepared you to address oral health topics during maternal and child health visits?

- If yes, how and in what ways?
- If no, what could we have done better?

1. What suggestions do you have for improving the training module for future cohorts of nurses and community health workers?

**Practicability of incorporation of oral health promotion activities into MCOH visits**

1. Since that you have completed your training on oral health promotion, how feasible do you think it is to incorporate oral health promotion activities into your routine maternal and child health visits regularly?

If feasible, how can you achieve this?

If not feasible, what challenges, have you encountered when trying to integrate oral health promotion into your visits?

1. Have you already started implementing any oral health promotion activities in your routine activities?

If yes, how soon after the training were you able to start providing oral health information to your patients?

What strategies have you used to engage and educate community members about oral health?

Follow up: how has it been received by your patients and the community?

1. Can you share any success stories or positive outcomes related to incorporating oral health promotion?

**Future directions**

1. Do you believe that the integration of oral health promotion into maternal and child health visits is sustainable in the long run?
2. What strategies or resources do you think are necessary to ensure the continued success of this initiative?
3. Are there any concerns about maintaining consistency in delivering oral health education over time?
4. How do you suggest monitoring and evaluating the impact of oral health promotion activities within your community?
